# Gender differences in the acceptance of wife-beating: evidence from 30 countries in Sub-Saharan Africa

**DOI:** 10.1186/s12905-023-02611-w

**Published:** 2023-08-27

**Authors:** Jones Arkoh Paintsil, Kenneth Setorwu Adde, Edward Kwabena Ameyaw, Kwamena Sekyi Dickson, Sanni Yaya

**Affiliations:** 1 Department of Economics, Howard University, Washington, USA; 2 Department of Population and Health, University of Cape Coast, Cape Coast, Ghana; 3 Institute of Policy Studies and School of Graduate Studies, Lingnan University, Tuen Mun, Hong Kong; 4 L & E Research Consult Ltd, Wa, Upper West Region, Ghana; 5 School of International Development and Global Studies, University of Ottawa, Ottawa, ON Canada; 6 The George Institute for Global Health, Imperial College London, London, UK

**Keywords:** Acceptance, Violence, Gender, Women, Intimate partner violence, Men sub-saharan Africa

## Abstract

**Background:**

The World Health Organization (WHO) has cited domestic violence as an urgent global maternal and child health priority. Gender differences in the acceptance of wife-beating have not been explored at the multi-country level in sub-Saharan Africa (SSA) where the occurrence of wife-beating (36%) is greater than the global average (30%). It is against this backdrop that we examine the gender differences in the acceptance of wife beating in SSA.

**Methods:**

We used Demographic and Health Survey data from 30 SSA countries. Acceptance of wife beating among women and men was the principal outcome variable of interest. We employed Multiple correspondence analysis and logistic regression model as the primary estimation methods for this study. The descriptive statistics show that women had a higher acceptance rate (44%) of wife beating than men (25%). For the women sample, Mali, Democratic Republic of Congo, Chad, and Guinea had higher rates of acceptance of the wife beating (80.6%, 78.4%, 77.1% and 70.3% respectively) For the men, only Guinea had an acceptance rate above 50 percent.

**Results:**

We found that all else equal, women’s acceptance of wife beating is higher for male-headed households than for female-headed households. Women without formal education were 3.1 times more likely to accept wife beating than those with higher education. Men with no formal education were 2.3 times more likely to accept wife beating than men with higher education. We found that polygamous women were comparable to polygamous men. Polygamous women were 1.2 times more likely to accept wife beating than those in monogamous marriages. Women were 1.2 times more likely to accept wives beating if they had extramarital relationships. Contrarily, men who engaged in extramarital affairs were 1.5 times more likely to justify wife beating. We also found that women’s acceptance of wife beating decreases as they age. Men who decide on major household purchases and spending decisions on their earnings are more likely to accept wife beating. Corollary, women with similar gender and employment roles also accept wife beating. Finally, exposure to mass media is significantly associated with lower acceptance of wife beating for women and men.

**Conclusion:**

We conclude that women have a higher acceptance rate of wife beating than men in SSA. Acceptance of wife beating differs significantly by country. Given the same level of education, women are more likely to accept wife beating than men. If women and men have similar levels of employment and gender roles, acceptance of wife beating is higher when men make major household purchasing decisions and and it is still higher even when the woman makes these decisions. Acceptance of wife beating is higher among young women and men, the uneducated, those in polygamous marriages, women, and men who engage in extra marital affairs, poor households and in rural areas. The findings indicate the need for policies and programs by SSA countries to truncate the high acceptance rate of wife beating, especially among women.

## Background

The World Health Organisation (WHO) cited domestic violence as an urgent global maternal and child health priority [1] and, estimated that, globally, one of every three women has experienced one or both physical and sexual violence [2] which amplifies the prevalence of domestic violence [3]. The WHO and Pan American Health Organization [4] described intimate partner violence (IPV) as any behaviour that ‘includes physical, sexual, and emotional abuse and controlling behaviours by an intimate partner’ (p.1). Wife beating on the other hand has been described as the physical aspect of IPV which includes hitting, beating, slapping, and kicking [1, 5].

IPV is an important human rights issue and of global public health concern which significantly influences socio-economic development [6]. Gashaw, Schel and Magnus [7] opined that the issue of IPV is a multifaceted phenomenon that is grounded in the interplay of society, community, family and at the individual level. For instance, studies have shown that gender role expectations are an important determinant of wife-beating [8–10]. Hence, cultures that foster beliefs of male superiority over women are more prone to wife-beating [8, 11]. Other studies have also found an association between wife beating and age, level of education, marital status, wealth index, place of residence, religion, occupation, previous experience of wife-beating, decision-making capacity, and level of media exposure [8, 9, 12–15].

Krause et al. [16] in their study observed that more women as compared to men justify wife beating. This phenomenon, Shrestha and Gartoulla [17] explained could be attributed to the entrenched belief of women that they deserve to be beaten for disobedience or for not living up to expectations. Particularly in patriarchal societies. Boris and Hughes [18] also attributed this phenomenon to the cultural and economic dependence of women on their husbands. This they argue forces women to tolerate wife beating. However, in cases where the women are financially dependent, Boris and Hughes [18] argued that wife beating is one of the tactics adopted by husbands to disrupt the woman’s economic activities and regain their dominance over the woman.

It is also worth noting that wife beating and any form of violence against women negatively affect the participation of women in societies politically, socially, and economically [18–20]. The utilisation of reproductive health services by women is also negatively affected by wife-beating [21–33]. Caykoylu et al. [24] also argued that wife beating does not only affect the woman (victim) but also affects children. Children with a history of exposure to IPV have a higher tendency of developing aggressive behaviour [24].

This paper examines the gender differences in acceptance of wife beating in Sub-Saharan Africa (SSA). This study is important for three reasons: First, acceptance of wife beating is still high in SSA [1, 25]. Second, acceptance of wife beating may lead to under-reporting of actual violence perpetrated against women as once women accept being abused, they are likely not to report. Third, studies [12–15, 26, 27] on the justification for wife beating have not focused on uncovering the gender differences in the acceptance of wife beating in SSA. Few studies have however examined the gender differences in the acceptance of wife-beating at country levels in Ghana [9], Uganda [15] and Nigeria [5]. However, this has not been explored at the multi-country level for sub-Saharan Africa (SSA) where the prevalence of wife-beating (36%) is greater than the global average of 30% [1, 25]. Uncovering the gendered pattern in acceptance of wife-beating will be an important driver in formulating national and international policies and intervention programmes geared towards alleviating wife-beating. It is against this backdrop that we examine the gender differences in the acceptance of wife beating in SSA.

## Methods

### Data source

We used data from the Demographic and Health Surveys (DHS) for the empirical investigation. For more than 30 years, USAID has been a pioneer in the Demographic and Health Survey Program. The DHS Program offers technical assistance to implement household-based surveys for developing countries across Asia, Africa, Latin America, and Eastern Europe. The DHS survey data naturally creates a hierarchy of the households within a cluster, the household members within each household, the interviewed women, and men as a subset of the household members, and the children of each interviewed Woman. The same person’s information may be gathered within this hierarchy via various questionnaires. For instance, data on women may be gathered through the Women Questionnaires as well as the Household Questionnaires. This is done similarly for the data collected on men. A typical DHS survey includes the population, household, women, men, child, and couple modules. Since the 1990s, the DHS has included questions about the justification for wife beating. To ensure objectivity and consistency of the analysis, we used the data to which information is available in the same survey period for women and men file. The women’s data set includes 123,117 sample between the ages of 15 and 49. In contrast, the men’s dataset included 75,975 sample between the ages of 15 and 54. Thus, the total number of SSA countries covered in the study to 30 and spans 2008 through 2020.

### Study variables and measurement

#### Outcome variable

The dependent variable of interest is acceptance/ justification of wife beating. The DHS has questions for both women and men module on whether the wife beating is justified if: (i) she goes out without telling the husband; (ii) she neglects the children; (iii) she argues with the husband; (iv) she refuses to have sex with the husband and (v) she burns the food. These variables are combined to form a dummy variable, coded one if the woman (man) accepts/justifies the wife beating and zero, otherwise.

#### Explanatory variables

Several studies at the country level examined determinates of wife beating [5, 9, 15]. The independent variables used in these studies can be grouped into three categories: women (men) characteristics (the current age of the respondent, education, employment, marriage type, extramarital sex, and media exposure) employment and gender roles (decision on major household purchases, decision on how to spend respondent’s earning, decision on respondent’s healthcare) and household characteristics (sex of household head, household wealth, type of place of residence). In line with studies [5, 9], current age has four categories-15-24, 25–34, 35–44 and 45 and above with 25–34 as the reference category; Education has four categories: no education, primary, secondary, and higher with higher as the reference category. We also controlled for the employment status coded one for currently working and zero for not working. Marital type is coded one for polygamous marriage and zero for monogamous marriage. Women (men) with extra marital affair is coded one for sex with one or more persons, else zero for sex with only partner. We also controlled for employment and gender roles; person who usually decides on large household purchases, person who usually decides how to spend respondent’s earnings and person who usually decides on respondent’s health care. We limited the responses to three categories—respondent alone, respondent and partner and partner alone with partner alone as the reference category. Further, we also controlled for mass media exposure, specifically, the frequency of watching television, reading newspapers, and listening to the radio. Each has three categories —not at all, at least once a week and almost every day with not at all as the reference category. The sex of the household head takes one for males and zero for females as the reference category. To account for women’s and men’s household wealth, we introduced the dummies of the wealth quintile; poorest, poorer, middle, richer and richest. Finally, we account for the type of place of residence coded one for rural and zero for urban.

### Data Analysis

We employed Multiple Correspondence Analysis (MCA) and logistic regression as the primary estimation approaches. We used MCA to check the overall fitness of the data. We applied the Burt method which performs a correspondence analysis of the Burt matrix of two-way cross-tabulations of all pairs of variables. Next, we used the logistic regression as the robust classification model in determining the possible outcome of our dependent variable—acceptance/justification for wife beating, given the explanatory variables. One key advantage of logistic regression is that the coefficients are easily interpreted and have high predictive power. We applied sample weight and cluster standard errors in all our estimations. We compute our estimates with STATA 17.

### Ethics approval

We did not require any additional ethical approval because we used publicly available secondary data for the analysis. Details of the ethical standards are available at http://goo.gl/ny8T6X.

## Results

### Study population characteristics

**Fig. 1:**
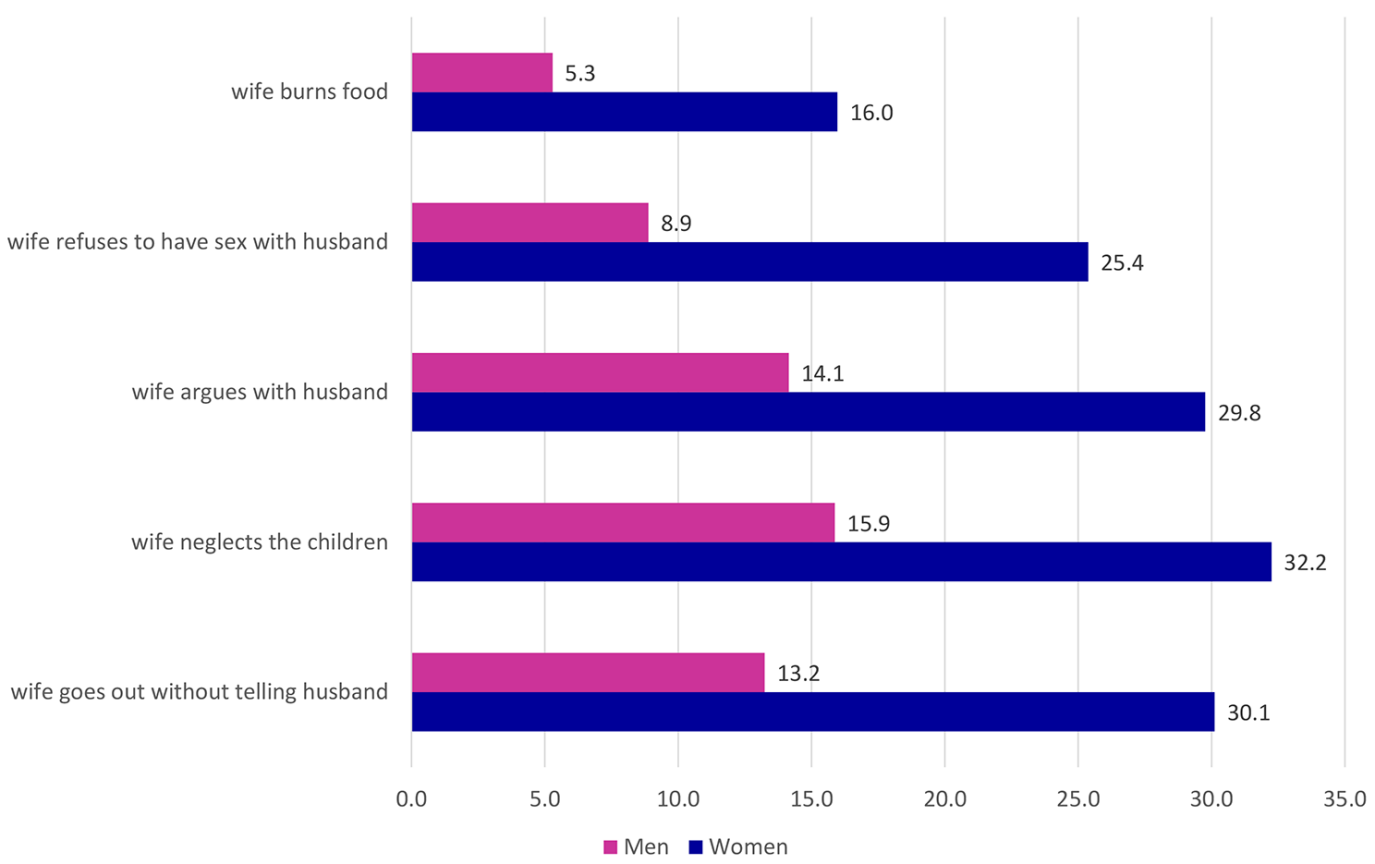
Reasons for justification of wife beating

Figure 1 shows the acceptance of wife’s beating by women and men. There are three notable points. First, women have a higher acceptance rate of wife’s beating than men in all categories. Second, the highest form of justification for wife beating is when a women neglect children but is about twice justifiable for women (32.2%) than men (15.9%). Third, the least acceptance of wife beating is when she burns the food and women acceptance is three times higher (16%) than men (5.3%).

**Fig. 2:**
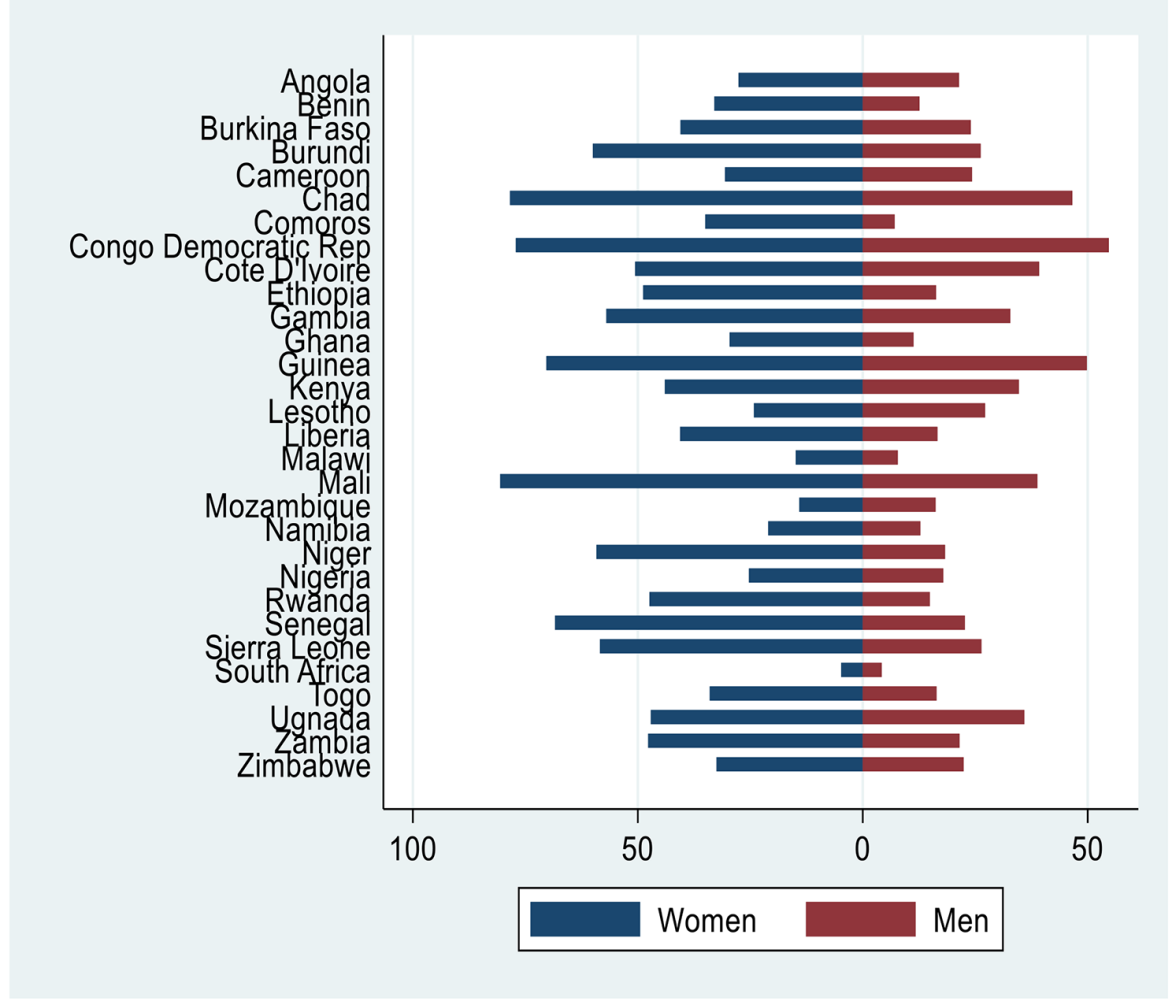
Justification of wife beating between women and men by country

Figure 2 shows the acceptance of wife-beating by country. This result shows that women in the sample usually accept wife beating as justified compared to men who perpetrate this violence. We also noted that the acceptance of wife beating differs by country. For the women sample, Mali, Chad, Congo Democratic Republic, and Guinea had the highest rate of acceptance (80.6%, 78.4%, 77.1% and 70.3%respectively). Only women in South Africa (4.9%) had an acceptance rate below 10%. For the men sample, Congo Democratic Republic (54.7%) had an acceptance rate above 50%. Unlike the women sample, three countries in the men sample had acceptance rates below 10% (South Africa, Comoros, and Malawi).

Next, we present the estimates from the MCA. The total principal inertia is approximately 0.05 for the women sample and 0.06 for the men sample. The first two dimensions explain about 70% and 56% of the total variation in the data for the women and men sample respectively. To be concise, Table 1 shows the mass, distance, inertia, and coordinates (dim1 & dim2) for the variables employed in the analysis. The statistics of interest are the inertia and coordinates points. There are key notable points. First, the coordinate points in the first dimension are dispersed for acceptance of wife beating, frequency of watching television, frequency of reading newspaper, frequency of listening to radio and decision on respondent earnings. This means that the responses to these questions by the women (men) were different. For example, women who accept wife beating had a negative coordinate compared to the positive coordinate of women who do not accept wife beating, women with higher education had positive coordinate versus the negative coordinate of those with no education. Second, we note that the responses in dimension two are dispersed around the zero coordinate. This means responses were similar in this dimension. Third, the response to sex of household head, age of the woman, working status, marital type, extra marital affair, purchase decision and health decision are located at the centre of zero coordinates in both dimensions which means there were no variations in the responses given by the women to these set of questions. Similar pattern is observed in the men sample such as the sex of the household head, age of the man, extra marital affair, and type of place of residence. Forth, the total inertia from each category of variables is similar for both the women and the men sample. Thus, the variables have similar overall pattern in the data.

**Table 1:** Multiple Correspondence Analysis

Categories	Mass	Women				Mass	Men			
		Distance	Inertia	dim1	dim2		Distance	Inertia	dim1	dim2
Wife beating										
No	0.037	0.260	0.001	0.758	0.050	0.050	0.156	0.000	0.320	0.062
Yes	0.029	0.329	0.001	-0.961	-0.063	0.016	0.475	0.000	-0.975	-0.188
Sex of head										
female	0.011	0.623	0.000	0.568	-1.255	0.004	1.089	0.000	0.644	0.168
male	0.056	0.119	0.000	-0.108	0.239	0.063	0.062	0.000	-0.037	-0.010
Age										
25–34	0.027	0.313	0.000	0.195	-0.006	0.022	0.376	0.000	0.079	0.086
15–24	0.011	0.591	0.000	-0.632	0.554	0.004	1.070	0.000	-0.713	0.712
35–44	0.021	0.385	0.000	0.119	-0.170	0.023	0.363	0.000	0.196	-0.102
45+	0.007	0.753	0.000	-0.094	-0.352	0.019	0.425	0.000	-0.186	-0.123
Education										
No education	0.025	0.423	0.002	-1.473	-0.468	0.016	0.568	0.002	-2.033	-0.508
primary	0.020	0.405	0.000	-0.299	0.984	0.021	0.402	0.000	-0.513	0.945
secondary	0.017	0.528	0.001	1.626	-0.275	0.022	0.399	0.001	0.985	-0.106
higher	0.004	1.199	0.002	3.734	-0.828	0.007	0.874	0.002	2.859	-1.259
Currently working										
no	0.004	0.985	0.000	-0.450	-0.017	0.002	1.538	0.000	-0.634	0.619
yes	0.062	0.069	0.000	0.031	0.001	0.065	0.044	0.000	0.018	-0.018
polygamy										
No	0.049	0.176	0.000	0.386	0.544	0.058	0.104	0.000	0.225	0.162
Yes	0.018	0.472	0.001	-1.036	-1.459	0.008	0.746	0.001	-1.604	-1.155
Extramarital affair										
No	0.065	0.035	0.000	-0.012	0.036	0.058	0.105	0.000	-0.061	0.091
Yes	0.001	1.941	0.000	0.645	-1.981	0.009	0.667	0.000	0.387	-0.582
Television										
Not at all	0.037	0.322	0.002	-1.231	0.706	0.029	0.389	0.002	-1.442	1.027
At least once a week	0.018	0.556	0.003	1.992	-1.102	0.023	0.466	0.002	1.586	-1.430
Almost everyday	0.002	1.803	0.002	2.617	-1.413	0.002	1.836	0.003	3.735	1.249
Newspaper										
Not at all	0.055	0.152	0.001	-0.572	0.120	0.043	0.249	0.001	-1.021	0.257
At least once a week	0.005	1.105	0.002	3.417	-0.994	0.012	0.676	0.002	2.403	-1.040
Almost everyday	0.000	5.699	0.000	4.122	-1.352	0.001	2.911	0.002	4.252	2.039
radio										
Not at all	0.025	0.391	0.001	-1.165	0.534	0.014	0.567	0.001	-1.509	1.012
At least once a week	0.026	0.365	0.001	0.949	-0.360	0.037	0.259	0.001	0.582	-0.556
Almost everyday	0.002	1.897	0.001	1.554	-0.936	0.002	1.615	0.002	2.533	1.685
Decision on large household purchases										
Respondent alone	0.010	0.679	0.001	0.279	-2.262	0.028	0.403	0.002	-1.131	-1.913
Respondent and partner	0.031	0.364	0.002	0.885	2.122	0.030	0.382	0.002	0.858	1.907
Partner alone/wife/husband	0.026	0.421	0.002	-1.164	-1.623	0.008	0.825	0.002	0.774	-0.299
Decision on respondent healthcare										
Respondent alone	0.014	0.576	0.001	0.580	-1.914	0.036	0.326	0.002	-0.791	-1.657
Respondent and partner	0.027	0.392	0.002	0.818	2.380	0.025	0.452	0.002	0.991	2.355
Partner alone/wife/husband	0.025	0.425	0.002	-1.193	-1.538	0.006	0.996	0.002	0.543	0.021
Decision on respondent earnings										
Respondent alone	0.037	0.255	0.000	-0.231	-1.317	0.034	0.353	0.002	-0.913	-1.823
Respondent and partner	0.021	0.458	0.001	0.943	2.665	0.028	0.412	0.002	0.978	2.161
Partner alone/wife/husband	0.008	0.743	0.001	-1.374	-0.879	0.005	1.174	0.002	0.621	-0.043
Wealth quintile										
poorest	0.012	0.641	0.001	-1.853	0.533	0.012	0.645	0.001	-1.996	0.934
poorer	0.012	0.591	0.001	-1.309	0.821	0.012	0.589	0.001	-1.248	0.835
middle	0.013	0.534	0.000	-0.542	0.358	0.013	0.542	0.000	-0.464	0.500
richer	0.014	0.523	0.000	0.550	-0.357	0.014	0.525	0.000	0.618	-0.374
richest	0.015	0.650	0.003	2.484	-1.068	0.016	0.608	0.003	2.277	-1.408
Type of place of residence										
Urban	0.026	0.438	0.002	1.646	-1.023	0.027	0.422	0.002	1.599	-1.182
Rural	0.041	0.282	0.002	-1.061	0.660	0.040	0.283	0.002	-1.074	0.794

### Differences in acceptance of wife beating between women and men

Table 2 presents the summary of the explanatory variables (in proportions) by acceptance of wife beating and tests whether the differences are significant. Among women whose spouses are heads of their households, acceptance of wife beating is higher (0.85), comparable to women who do not accept it (0.83). This is comparable to men’s sample where male-headed household heads turn to accept wife beating than those who do not. Thus, all else equal, women’s acceptance of wife beating is higher for male-headed households than female-headed households. We note that younger women (15–24 years) turn to accept wife beating and this is equivalent to men in the same category who justify wife beating. Women within the age group 25–34 years have a lower acceptance to wife beating (0.40). Contrarily, men within the same group have higher acceptance of wife beating. On average, acceptance of wife beating decreases with age for both women and men. We note that educational attainment makes a difference in acceptance of wife beating. Specifically, acceptance of wife beating is higher for no education and primary education for both women and men. Conversely, secondary and higher education levels have lower acceptance of wife beating for both women and men.

On average, acceptance of wife beating is higher in polygamous marriages and men with extra marital sex partners justify wife beating in a higher proportion with respect to employment and gender roles, we note that if the men make major household purchases, the difference (those who do not accept versus those who accept wife beating) is about 12% higher but for women it is about 0.4%. Conversely, if the roles are shared by both the woman and the partner, the difference in acceptance of wife beating is about 13% lower. We note that if the women have greater say in their health decisions, acceptance of wife beating is lower but for men, acceptance of wife beating is higher. However, if health care decision is taken by both the woman and the man, acceptance of wife beating is lower. The difference in acceptance of wife beating is about 1% lower if women usually decide how to spend their earnings but for men it is about 11% higher. Exposure to mass media (television, newspaper, and radio) lowers the acceptance of wife beating for both women and men. We note that belonging to a poor household is associated with higher acceptance of wife beating compared to wealthy households. All things being equal, women in rural areas have higher acceptance of wife beating for both women and men.

**Table 2:** The difference in acceptance of IPV by covariates

	Women			p-value	Men			p-value
	Not accept	Accept	Difference		Not accept	Accept	Difference	
**Male household head**	0.829	0.855	-0.027	0.000	0.944	0.952	-0.009	0.000
**Age**								
15–24	0.148	0.196	-0.047	0.000	0.050	0.077	-0.026	0.000
25–34	0.413	0.404	0.009	0.001	0.315	0.361	-0.045	0.000
35–44	0.327	0.299	0.028	0.000	0.344	0.322	0.022	0.000
45+	0.112	0.101	0.010	0.000	0.290	0.241	0.050	0.000
**Education**								
No education	0.305	0.472	-0.167	0.000	0.222	0.292	-0.070	0.000
Primary	0.287	0.326	-0.039	0.000	0.303	0.347	-0.044	0.000
Secondary	0.309	0.184	0.125	0.000	0.346	0.310	0.036	0.000
Higher	0.099	0.018	0.080	0.000	0.130	0.051	0.078	0.000
**Currently working**	0.943	0.925	0.018	0.000	0.973	0.969	0.004	0.004
**Polygamous**	0.234	0.319	-0.085	0.000	0.114	0.151	-0.037	0.000
**Extra marital affair**	0.018	0.018	0.000	0.779	0.125	0.168	-0.043	0.000
**Decision on purchases**								
Respondent alone	0.152	0.156	-0.004	0.047	0.396	0.518	-0.122	0.000
Respondent and husband/partner	0.515	0.390	0.125	0.000	0.474	0.366	0.109	0.000
Partner alone	0.333	0.454	-0.121	0.000	0.130	0.116	0.014	0.000
**Decision on heath care**								
Respondent alone	0.222	0.185	0.037	0.000	0.505	0.616	-0.111	0.000
Respondent and husband/partner	0.463	0.348	0.115	0.000	0.398	0.300	0.099	0.000
Partner alone	0.315	0.468	-0.153	0.000	0.096	0.084	0.012	0.000
**Decision on earnings**								
Respondent alone	0.554	0.566	-0.011	0.000	0.477	0.585	-0.108	0.000
Respondent and husband/partner	0.348	0.278	0.071	0.000	0.451	0.352	0.099	0.000
Partner alone	0.097	0.157	-0.060	0.000	0.071	0.062	0.009	0.000
**Television**								
Not at all	0.474	0.655	-0.181	0.000	0.409	0.513	-0.104	0.000
At least once a week	0.161	0.138	0.023	0.000	0.199	0.197	0.002	0.546
Almost every day	0.344	0.198	0.146	0.000	0.371	0.280	0.091	0.000
**News paper**								
Not at all	0.777	0.900	-0.124	0.000	0.629	0.722	-0.094	0.000
At least once a week	0.124	0.064	0.061	0.000	0.172	0.143	0.029	0.000
Almost every day	0.097	0.035	0.062	0.000	0.193	0.132	0.061	0.000
**Radio**								
Not at all	0.334	0.431	-0.097	0.000	0.201	0.255	-0.054	0.000
At least once a week	0.226	0.206	0.020	0.000	0.196	0.206	-0.010	0.003
Almost every day	0.422	0.349	0.072	0.000	0.577	0.520	0.057	0.000
**Wealth quintile**								
Poorest	0.135	0.240	-0.105	0.000	0.160	0.241	-0.081	0.000
Poorer	0.160	0.221	-0.061	0.000	0.173	0.214	-0.041	0.000
Middle	0.187	0.208	-0.021	0.000	0.186	0.206	-0.020	0.000
Richer	0.217	0.190	0.027	0.000	0.207	0.192	0.015	0.000
**Rural**	0.532	0.705	-0.173	0.000	0.569	0.686	-0.116	0.000
Overall acceptance of wife beating		0.441				0.247		
N	123,117				75,975			

### Covariates of acceptance of IPV for women and men

Table 3 presents the logistic regression result of women’s and men’s acceptance of wife beating. An important question we answered here is whether acceptance of wife beating vary significantly between women and men. We found that all else equal, women’s acceptance of wife beating is higher for male-headed households than for female-headed households, and the result is statistically significant at 1%. This result also holds for the men’s sample at 5% significance. Acceptance of wife beating increases with young women (15–24) compared to women between the ages 25–34 years. As age increase beyond 35 years, acceptance of wife beating falls, and this is also consistent with men. Women with no education were 3.1 times more likely to accept wife beating than women with higher education.

However, for men with no education, the odds were 2.3 times. Women with primary and secondary education were 2.8 times and 2.1 times more likely to accept wife-beating. Men with primary and secondary education were 2.1 times and 1.7 times more likely to accept wife beating. This result suggests that, given the same level of education, justification for wife beating is higher for women than men. Being employed decreases the odds of accepting wife beating for women and is insignificant for men. We found that women in polygamous marriages were 1.2 times more likely to accept wife beating than women in monogamous marriages, which is comparable to men who have multiple wives (1.2 times). The odds of women who engaged in extramarital affairs were 1.2 times more likely to accept wife beating. In contrast, men who engaged in extramarital were 1.5 times more likely to justify wife-beating. With reference to employment and gender roles, we find that when women decide major household purchases alone, justification for wife beating is higher when compared to when husband makes the decision alone. This is also true when both the woman and partner decide on major household purchases together.

With respect to the men’s sample, we note that the acceptance of wife beating is even higher when the man decides major household purchases and when the man makes the decision with the wife. We found that when the woman makes healthcare decisions with the husband, acceptance of wife beating is higher compared to when the husband makes the decision alone. For men, acceptance of wife beating is higher when the man makes the healthcare decision alone compared to when the wife makes the health decision alone for the husband. This finding also holds when the woman decides how to spend her earnings alone and when her spending decision is made with the husband. Women who frequently watched television and read newspapers almost every day had lower odds of accepting wife beating than women who do not. This result holds for men who read newspapers almost daily. Again, we found that belonging to households with less family wealth is associated with higher odds of acceptance of wife beating for both women and men. For example, women and men from the poorest and poorer wealth quintiles, respectively, had 2.2 times and 1.9 times more likely to accept wife beating when compared with those from the richest wealth quintile. Finally, we found that living in a rural area is associated with higher odds of acceptance of wife beating for women in rural areas, and this result also holds for men.

**Table 3:** Binary logistic regression of acceptance of IPV for women and men

VARIABLES	Women		Men	
	Odds ratio	CI	Odds ratio	CI
Sex of head				
Male	1.107***	(1.062–1.153)	1.098**	(1.010–1.194)
Current age				
15–24	1.148***	(1.104–1.193)	1.212***	(1.121–1.310)
35–44	0.909***	(0.881–0.938)	0.847***	(0.810–0.885)
45+	0.909***	(0.868–0.951)	0.704***	(0.671–0.738)
Education				
No education	3.147***	(2.863–3.459)	2.325***	(2.110–2.562)
Primary	2.831***	(2.592–3.093)	2.108***	(1.929–2.304)
Secondary	2.140***	(1.972–2.323)	1.687***	(1.558–1.827)
Currently working				
Yes	0.934**	(0.875–0.996)	0.981	(0.878–1.096)
Marriage type				
Polygamy	1.222***	(1.180–1.265)	1.167***	(1.106–1.232)
Extra marital affairs				
Yes	1.192***	(1.069–1.331)	1.533***	(1.452–1.619)
Decision of large household purchases				
Respondent alone	1.160***	(1.099–1.225)	1.279***	(1.202–1.362)
Husband/partner alone	1.163***	(1.109–1.219)	1.225***	(1.130–1.327)
Decision of respondent’s health care				
Respondent alone	1.006	(0.962–1.051)	1.152***	(1.082–1.226)
Husband/partner alone	1.306***	(1.248–1.367)	0.978	(0.881–1.085)
Decision on respondent’s earnings				
Respondent alone	1.075***	(1.026–1.126)	1.121***	(1.048–1.199)
Husband/partner alone	1.197***	(1.126–1.273)	0.987	(0.890–1.096)
Frequency of watching television				
At least once a week	0.982	(0.937–1.029)	1.032	(0.975–1.092)
Almost everyday	0.911***	(0.869–0.955)	1.015	(0.955–1.078)
Frequency of reading newspaper				
At least once a week	0.868***	(0.825–0.914)	0.976	(0.921–1.035)
Almost everyday	0.832***	(0.778–0.891)	0.865***	(0.809–0.924)
Frequency of listening of radio				
At least once a week	1.031	(0.987–1.078)	1.043	(0.986–1.104)
Almost everyday	1.027	(0.983–1.073)	0.993	(0.943–1.046)
Wealth quintile				
Poorest	2.148***	(1.992–2.317)	1.892***	(1.731–2.069)
Poorer	1.812***	(1.694–1.938)	1.605***	(1.473–1.748)
Middle	1.605***	(1.509–1.709)	1.524***	(1.409–1.649)
Richer	1.430***	(1.361–1.503)	1.355***	(1.266–1.450)
Type of place of residence				
Rural	1.074**	(1.016–1.136)	1.069**	(1.007–1.136)
Constant	0.070***	(0.059–0.082)	0.073***	(0.058–0.091)
Country fixed effect	Yes		Yes	
Observations	123,117		75,975	
Pseudo R2	0.161		0.108	

The reference category female household head, respondent with age 25–34 years, Woman (man) with highest, not working, monogamy, no extra marital affairs, partner alone, not at all, richest wealth quintile, urban. *** p < 0.01, ** p < 0.05, * p < 0.1

## Discussion

We explored the gender differences in wife beating in SSA. The acceptance of wife beating was highest in Mali and lowest in South Africa for women. For the men, the acceptance rate was highest in Democratic Republic of Congo and lowest in South Africa. We found that women have a higher acceptance rate of wife beating than men. These findings affirm previous studies’ results [27–29]. We assert this observation to the patriarchal bargaining theory [30], which contends that women living in traditional patriarchal societies accept the standard of blaming the wife in cases of wife beating. Women in patriarchal settings also view wife-beating as punishment for their wife’s disobedience rather than violent behaviour [29]. We also observed that all things being equal, women’s acceptance of wife beating was higher in male-headed households than in female-headed households. This finding further explains the argument for the role of the patriarchal system in enforcing wife-beating. This suggests the need for advocacy and education of women in patriarchal societies to sensitise them on the difference in discipline and domestic violence.

We also found that women and men who accept wife-beating is associated with young age as compared to those who do not accept wife beating. This conforms to the findings of previous studies that also observed that acceptance of wife beating was higher among younger women [31–33]. A plausible explanation is that as people age, they get more exposure and an incredible social network that helps them better understand wife beating and domestic violence [31]. This exposure thus helps women make an informed decision on what constitutes discipline and abuse. Likewise, in the present study, an increase in the level of education also showed a decrease in the acceptance of wife beating. This affirms the studies of Erten and Keskin [34] and Andarge et al. [31], who argued that education positively impacts knowledge of domestic violence. Plausibly, this enabled people to disprove their early socialisation of wife-beating acceptance. We believe this strongly indicates the need for policies and programmes to promote education. It is worth noting the present study revealed that given the same level of education, women are more likely to accept wife beating than men. This suggests the need for targeted intervention for women to budge their socialisation to accept wife beating.

Regarding the influence of media on the acceptance of wife beating, we found that both women and men who frequently watch television and read the newspaper are less likely to accept wife beating. This evidence corroborates the findings of previous studies that also found that exposure to mass media reduces the likelihood of accepting wife-beating [9, 35]. The importance of mass media in reducing the incidence of wife-beating [36] is, thus, paramount. Like previous studies, [5, 37, 38], men and women in polygamous marriages are likelier to accept wife beating than their counterparts in monogamous marriages. Amo-Adjei and Tuoyire [38] argued that women in polygamous marriages are likely to be of poor socioeconomic status. Consequently, they must accept wife beating and other social conventions to keep their marriage intact.

As reported by some studies [5, 9], the likelihood of accepting wife beating is higher when wife makes all the decisions regarding major household purchases and spending on her earnings. Acceptance of wife beating is much higher when the husband is the sole decision maker for major household purchases and his healthcare. No evidence exists when the woman makes the healthcare decision alone and when the husband makes healthcare decision with the wife. Bloch and Rao [39] and Bulte and Lensink [40] found that women’s employment status positively correlates with wife beating. They argued that men married to economically empowered women in developing countries use violence to keep their wives submissive [41]. We, however, found a contradictory result from the above. The study found a significant difference between currently employed women who accept IPV versus those who do not. Expressly, employed women reject IPV more than those who accept it. We attribute this finding to the fact that gainfully employed women have better bargaining power and will not tolerate intimate partner violence [42]. We further found that belonging to a poor household is associated with higher acceptance of wife beating than their wealthy counterparts. This result conforms to the findings of previous studies [9, 43]. A possible explanation could be that women in poor households are likely to be undereducated and less empowered and hence are more likely to accept wife-beating [31]. The depression and frustration of men’s inability to provide for their families could make men in poorer families more likely to accept wife-beating [9, 43]. Sardinha and Catalan [29] also argued that countries with more gender-equitable economic rights experience lower acceptance of domestic violence. Therefore, SSA countries must create an environment that promotes gender-equitable economic rights to improve the financial status of households.

### Strength and limitations

This study used a strict analytical methodology to examine gender differences in acceptance of wife beating in SSA. We drew our sample from 75,975 men and 123,117 women in 30 SSA countries from 2008 to 2020, demonstrating the validity and generalisability of our findings. The study does have a significant shortcoming, though. Thus, since this study is cross-sectional, it is impossible to draw any causal inference between gender differences and the acceptance of wife beating.

## Conclusion

We examined the gender differences in the acceptance of wife beating in SSA. We found that women have a higher acceptance rate of wife beating than men. We also found that the acceptance of wife beating differs by country. Mali recorded the highest acceptance rate for women, while South Africa recorded the lowest. Cote d’Ivoire recorded the highest acceptance rate for men, while South Africa recorded the lowest. Sex of the household head, age, level of education, type of marriage, extramarital affairs, watching television, listening to the radio, reading newspapers, wealth status, and place of residence had significant associations with the acceptance of wife beating for both men and women. The findings from this study indicate the need for policies and programs by SSA countries to truncate the high acceptance rate of wife beating, especially among women.

## Data Availability

Data for this study were sourced from Demographic and Health surveys (DHS) and available here: http://dhsprogram.com/data/available-datasets.cfm.
